# Quantification of Rapid Myosin Regulatory Light Chain Phosphorylation Using High-Throughput In-Cell Western Assays: Comparison to Western Immunoblots

**DOI:** 10.1371/journal.pone.0009965

**Published:** 2010-04-01

**Authors:** Hector N. Aguilar, Barbara Zielnik, Curtis N. Tracey, Bryan F. Mitchell

**Affiliations:** 1 Department of Physiology, Faculty of Medicine and Dentistry, University of Alberta, Edmonton, Alberta, Canada; 2 Department of Obstetrics and Gynecology, Faculty of Medicine and Dentistry, University of Alberta, Edmonton, Alberta, Canada; Universidad Europea de Madrid, Spain

## Abstract

**Background:**

Quantification of phospho-proteins (PPs) is crucial when studying cellular signaling pathways. Western immunoblotting (WB) is commonly used for the measurement of relative levels of signaling intermediates in experimental samples. However, WB is in general a labour-intensive and low-throughput technique. Because of variability in protein yield and phospho-signal preservation during protein harvesting, and potential loss of antigen during protein transfer, WB provides only semi-quantitative data. By comparison, the “in-cell western” (ICW) technique has high-throughput capacity and requires less extensive sample preparation. Thus, we compared the ICW technique to WB for measuring phosphorylated myosin regulatory light chain (PMLC_20_) in primary cultures of uterine myocytes to assess their relative specificity, sensitivity, precision, and quantification of biologically relevant responses.

**Methodology/Principal Findings:**

ICWs are cell-based microplate assays for quantification of protein targets in their cellular context. ICWs utilize a two-channel infrared (IR) scanner (Odyssey®) to quantify signals arising from near-infrared (NIR) fluorophores conjugated to secondary antibodies. One channel is dedicated to measuring the protein of interest and the second is used for data normalization of the signal in each well of the microplate. Using uterine myocytes, we assessed oxytocin (OT)-stimulated MLC_20_ phosphorylation measured by ICW and WB, both using NIR fluorescence. ICW and WB data were comparable regarding signal linearity, signal specificity, and time course of phosphorylation response to OT.

**Conclusion/Significance:**

ICW and WB yield comparable biological data. The advantages of ICW over WB are its high-throughput capacity, improved precision, and reduced sample preparation requirements. ICW might provide better sensitivity and precision with low-quantity samples or for protocols requiring large numbers of samples. These features make the ICW technique an excellent tool for the study of phosphorylation endpoints. However, the drawbacks of ICW include the need for a cell culture format and the lack of utility where protein purification, concentration or stoichiometric analyses are required.

## Introduction

Western immunoblotting (WB) is widely utilized to study relative levels of signaling intermediates including a variety of phospho-proteins (PPs). The increasing availability of antibodies for specific PPs has enhanced the popularity of this relatively inexpensive technique. Recently, there has been increasing use of near-infrared (NIR) fluorophore–conjugated antibodies for the WB technique. Compared to chemiluminescent antigen detection, NIR fluorophores extended the linear range of detection and potentially improve WB sensitivity. [1], [2] These two properties improve quantification of highly abundant or relatively scarce proteins in cell lysates. Currently available NIR scanners (Odyssey®) for this purpose have two separate channels. This enables simultaneous detection of two different proteins, providing that the primary antibodies are raised in different species and that the species-specific secondary antibodies are labeled with different fluorophores. This segregation of signals is particularly useful in phosphorylation studies since it facilitates normalization of the PP signal to that of the total protein. However, WB continues to be a low-throughput, labour intensive technique. An additional consideration for the assessment of PPs is the potential for excessive variability particularly during protein transfer from the gel to the membrane, which might diminish the precision of the assay. [3], [4]

The in-cell-western (ICW) technique is a cell-based assay for the measurement of proteins in their cellular context. ICWs utilize 96-well or 384-well microplates into which adherent or non-adherent cells can be plated and analyzed using the Odyssey® scanner. This methodology requires the segregation of signals derived from the protein of interest (POI) and a normalization signal (reference protein [actin, glyceraldehyde-3-phosphate-dehydrogenase [GAPDH], etc.], or total cell content) into one of the two detection channels. ICWs eliminate the need for protein harvesting, lysate preparation, electrophoretic separation and electrophoretic transfer steps. However, due to the absence of a protein separation step, ICWs require that primary antibodies be highly specific for the POIs in the context of microscopy. Currently, only a few reports contain data produced by ICWs, and none of these include thorough validations of the ICW technique beyond evaluating antibody specificity using WB. [5], [6], [7], [8], [9] In this work, we attempted a more thorough technical evaluation of ICWs using NIR fluorescence-based WBs as a reference standard, thus eliminating the signal detection methodology as a confounding factor in comparing the two techniques. Further, we evaluated the ability of both techniques to measure a phosphorylation event in response to a physiological stimulus. Our endpoint of choice was the phosphorylation of myosin regulatory light chain (MLC_20_) in cultured human uterine myocytes. Phosphorylation of MLC_20_ at Ser^19^ in smooth muscle cells is a well characterized event that permits the myosin(II)-actin cross-bridging and is the hallmark biochemical event leading to tissue-level force production. We therefore monitored phospho(Ser^19^)-MLC_20_ (PMLC_20_) formation in cultured uterine myocytes stimulated with oxytocin (OT), the most potent biological stimulant for this cell type.

This work addressed two specific aims: 1) to assess and compare the linearity, specificity and precision of signals measurable by WB and ICW, and 2) to evaluate the ability of ICWs to yield comparable data to WB regarding concentration- and time-dependency of physiological phosphorylation events. Our broader goal was to determine the suitability of ICW as a technique that may be substituted for WB in signaling studies, particularly for high-throughput experimental protocols.

## Results

### Signal Linearity and Specificity for WB and ICW

**Figure 1:**
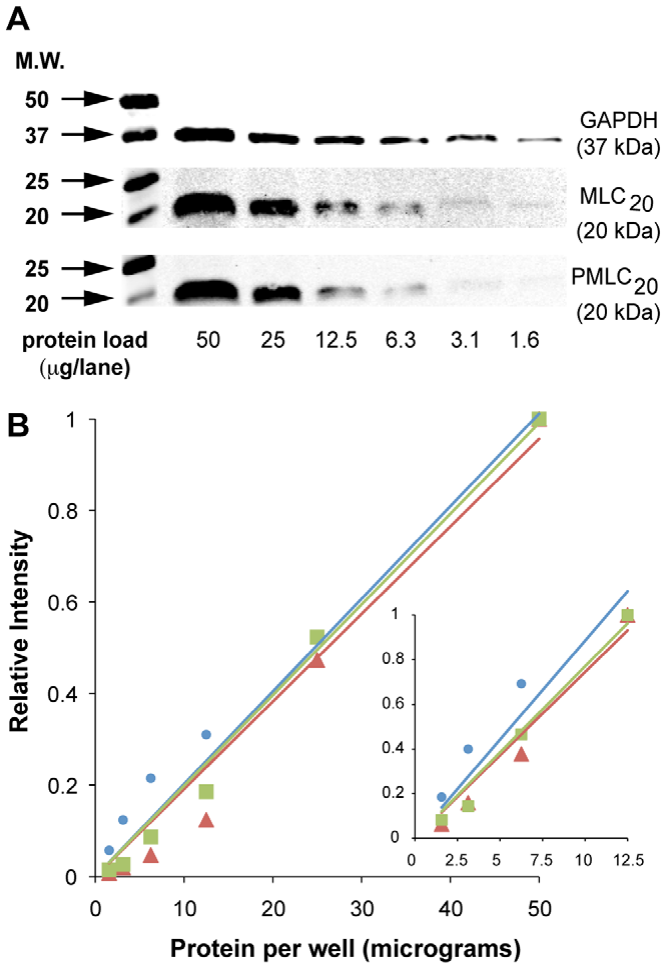
Signal linearity with western blots. **A.** Tris-Glycine-SDS gels were loaded with total uterine myocyte lysate (2-fold dilutions from 50 µg to 1.6 µg). GAPDH, total MLC20, and phospho(Ser19)-MLC20 (PMLC20) were detectable with 1.6 µg total protein/lane. **B.** Quantification of bands shown in panel A. Normalized values are expressed as a fraction of the band intensity measured at 50 µg/lane. Fit of a linear model through the origin showed excellent correlation for all three proteins (GAPDH: •; MLC20: ▪; PMLC20: ▴). The inset graph illustrates the correlation coefficient when only the lanes with ≤12.5 µg protein are analyzed. Molecular weight markers (MW) are shown at the left.

We assessed the linearity of both phosphorylated (PMLC_20_) and non-phosphorylated (GAPDH, total MLC_20_) signals via WBs prepared using 2-fold serial dilutions of uterine myocyte lysates starting at 50 µg total protein/lane. Preliminary experiments measuring GAPDH reached a lower limit of 0.19 µg protein/lane and showed that WB could not discriminate between protein loads ≤1.6 µg/lane. Therefore 1.6 µg/lane was selected as the lowest load for subsequent experiments. Of four replicate membranes prepared simultaneously to measure GAPDH, three of these revealed a good ability to discriminate antigen signal intensities. However, the signals from the fourth membrane could not discriminate bands in lanes loaded with less than 12.5 µg protein/well. The data from representative membranes for signals from GAPDH, PMLC_20_ and total MLC_20_ ranging from 50 to 1.6 µg/lane are shown in Figure 1. Though all three proteins may be quantified above 50 µg protein/lane (lane 1) in our experience, loading Tris-Glycine minigels with greater protein loads may decrease band resolution. The high correlation coefficients over this range for GAPDH, total MLC_20_, and PMLC_20_ (0.97, 0.99, 0.97, respectively: Figure 1B) support the conclusion that this technique can detect changes in specific protein concentrations. Even when the analysis is restricted to protein applications less than ≤12.5 µg/lane (Figure 1B inset), the correlation coefficients remain high for GAPDH (0.87) total MLC_20_ (0.98) and PMLC_20_ (0.96).

**Figure 2:**
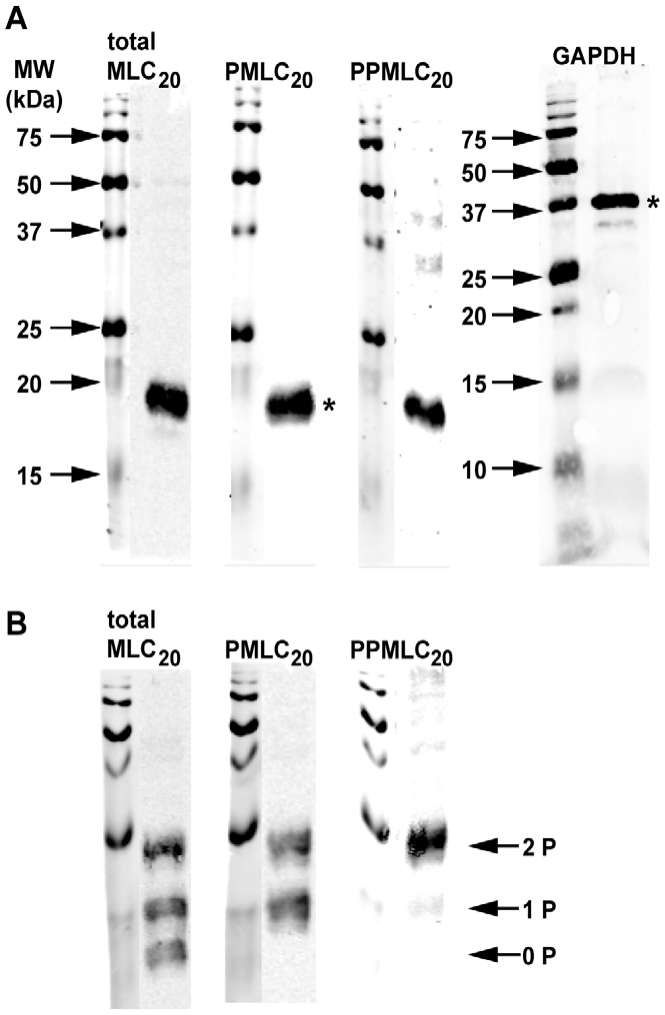
Antibody specificity using western blots. **A.** Full length western blots with single lanes loaded with 25 µg of protein/lane demonstrating the relevant bands (*) used for quantification in other experiments. Antibodies against GAPDH, total MLC_20_, PMLC_20_ and diphospho-MLC_20_ (PPMLC_20_) each identified one prominent band, though faint “non-specific” bands appeared with the anti-GAPDH and anti-PPMLC_20_ antibodies. **B.** Western blots performed as in panel A but with the addition of phos-tag (30 µM) to the gel matrix to promote mobility shifts in phosphorylated proteins. Probing for total MLC_20_ reveals 3 bands corresponding to unphosphorylated (0 P), mono-phosphorylated (1 P), and diphosphorylated (2 P) MLC_20_. The anti-PMLC_20_ antibody reacts with mono- and diphosphorylated MLC_20_ and fails to recognize the unphosphorylated species. The anti-PPMLC_20_ antibody recognizes primarily diphosphorylated MLC_20_ and demonstrates almost no cross-reactivity with the unphosphorylated or mono-phosphorylated MLC_20_. Molecular weight markers (MW) are shown to the left of each blot.

The specificity of the antibodies was assessed in Figure 2. The full length WB demonstrates the expected prominent bands relative to the molecular weight (MW) marker for each of the proteins (Figure 2A). Though bands of different sizes are detectable at several other positions, their relative intensities compared to the predominant bands are considered quantitatively insignificant (<5% signal relative to the band of interest). Probing with anti-GAPDH and anti-PMLC_20_ antibodies simultaneously (as performed for subsequent concentration- and time-response experiments) revealed two clear and prominent bands at the correct MWs (not shown). Introduction of 30 µM phos-tag into the polyacrylamide matrix markedly enhances separation of phosphorylated proteins by retarding their movement. [10] Our data (Figure 2B) demonstrate that the anti-PMLC_20_ antibody recognizes only the phosphorylated (mono- and di-) MLC_20_ species, in contrast to the anti-total MLC_20_ antibody, which reveals the unphosphorylated form as well as the mono- and diphospho-MLC_20_ (PPMLC_20_). For reference, the anti-PPMLC_20_ antibody identifies only the uppermost band in these last three panels, which corresponds to PPMLC_20_. These results confirm those in a previous study and indicate the highly specific nature of these antibodies. [11]

**Figure 3:**
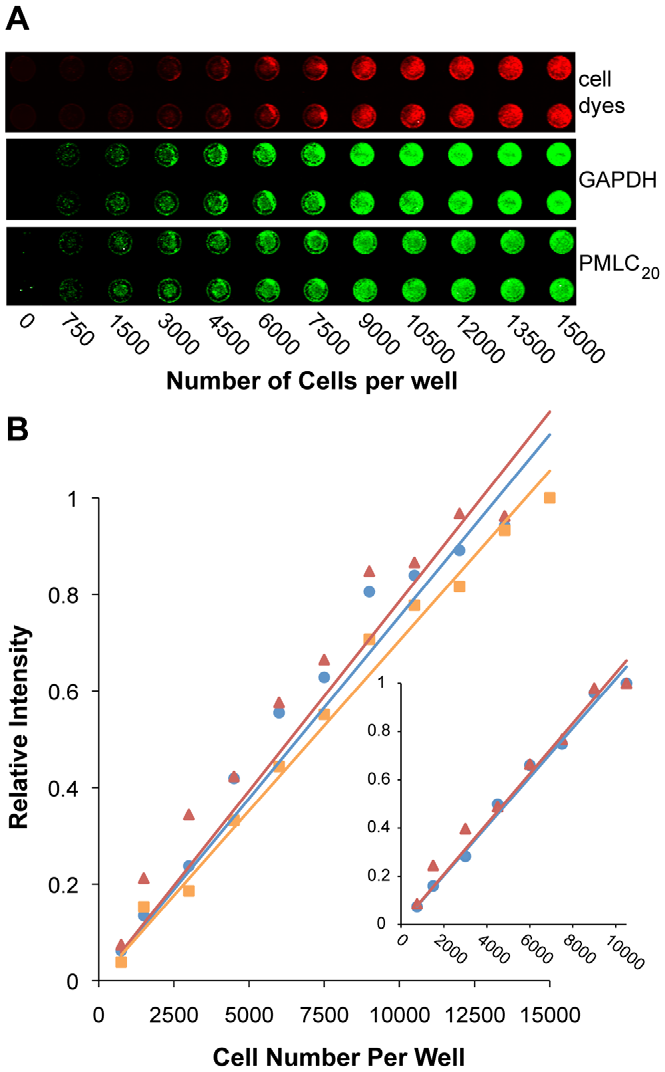
Signal linearity with the in-cell western assay. **A.** 96-well microplates were loaded with an increasing number of cells/well. Signals from anti-GAPDH and anti-PMLC_20_ antibodies appear as green fluorophores. Signals from cell dyes (cell number normalization) appear as red fluorophores. **B.** Quantification of signals shown in panel A. Normalized values are expressed as a fraction of the signal intensity of the wells containing 15,000 cells for GAPDH (•), PMLC_20_ (▴). The signal from the cell dyes also is shown (▪). The GAPDH and PMLC_20_ signals appear to plateau at the higher cell densities so the line of best fit has been calculated and illustrated by expressing the normalized values as a fraction of the intensity of the wells containing 10,500 cells (inset) for GAPDH (•), and PMLC_20_ (▴).

We assessed the linearity between signal intensity and cell number for ICWs using the antibody to PMLC_20_, and two normalization references (Figure 3). The first normalization reference used an antibody toward GAPDH. The second utilized a combination of two cell dyes (DRAQ5 [12] and Sapphire700, referred to as “cell dyes” in this manuscript) that provide an estimate of the cell content of the well and is detected in a separate channel of the IR scanner. Uterine myocytes were seeded into 96-well plates (15 mm^2^/well) from 750 cells/well through to 15,000 cells/well, resulting in adherent monolayers corresponding to cell densities of 0, 50, 100, 200, 300, 400, 500, 600, 700, 800, 900, and 1000 cells/mm^2^ (Figure 3A). Signals were detectable above background readings (empty wells) down to 750 cells/well. It was apparent that the relationship between cell numbers and concentrations of PMLC_20_ and GAPDH (Figure 3B) was plateauing at cell numbers beyond 10,500/well (700 cells/mm^2^). Using only the cells with equal to or less than cell density 700 cells/mm^2^ (inset), the correlation coefficients to a linear model for both GAPDH (0.99) and PMLC_20_ (0.96) were very high, supporting the conclusion that this assay has precision to measure changes in protein concentrations with optimal performance using 1,000–10,500 cells/well, which corresponds to 0.3 to 4 µg protein/well, based on average lysate yields for this cell type. Further, these data suggest that ICWs exhibit improved sensitivity compared to WB as we reliably measured GAPDH in ICWs below the lower limit of signal discrimination (1.6 µg/lane) by WB.

**Figure 4:**
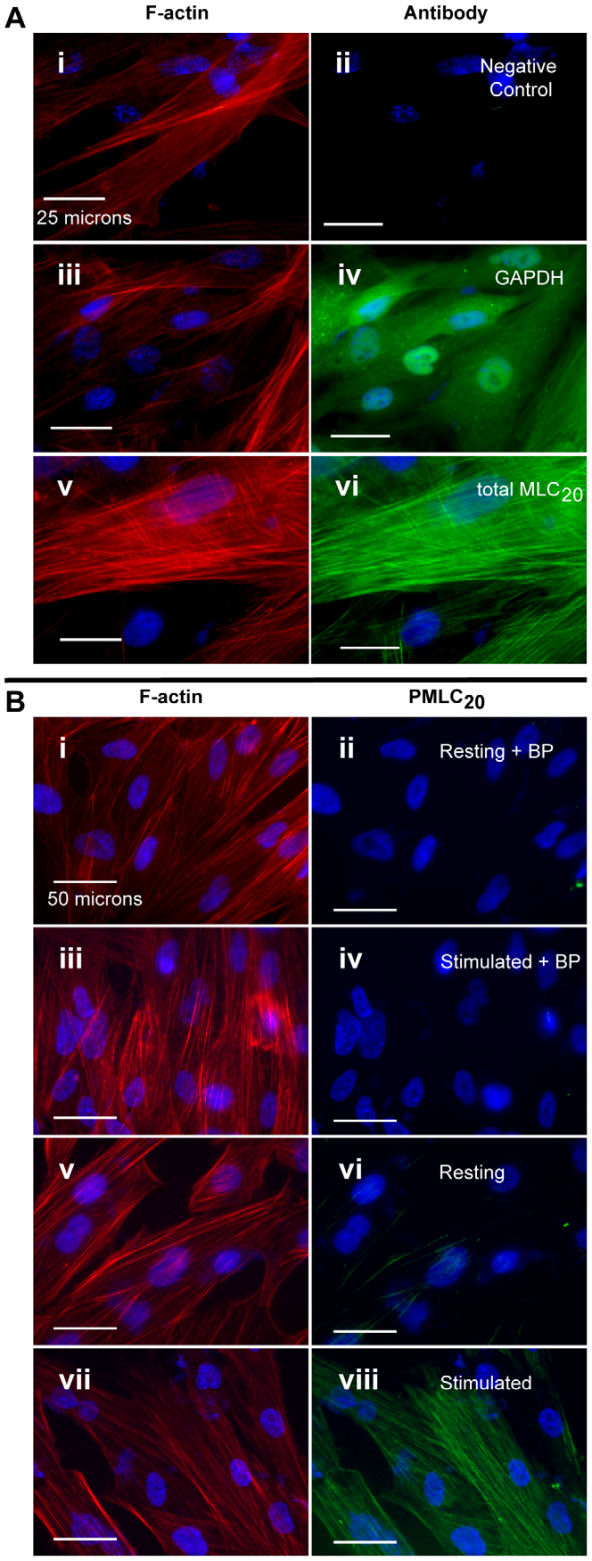
Antibody specificity using the in-cell western assay. In each pair of micrographs, the left panel illustrates filamentous actin (F-actin) stained with rhodamine-phalloidin (red). The corresponding right panels are immunofluorescence micrographs stained with antibodies conjugated to Alexa-Fluor 488 (green). Nuclei are stained with DAPI (blue) in all panels. Images are shown at 400× and 200× magnification in panels A and B, respectively. White bars represent 25 and 50 microns, respectively. **A.** Demonstration of GAPDH and total MLC_20_. Panels i and ii. The actin fibers stain in a filamentous pattern typical of uterine smooth muscle. There is no detectable signal with omission of the primary antibodies. Panels iii and iv. The GAPDH staining shows a diffuse cytosolic pattern in contrast to the fibrillar pattern of actin. Panels v and vi. Total MLC has a similar staining pattern to actin. **B.** Demonstration of PMLC_20_. Panels i–iv. There is no detectable background fluorescence when the antibody has been preadsorbed with blocking peptide (BP) containing phospho-Ser^19^ of MLC_20_, either in the resting state (panel ii) or with stimulation using 100 nM OT (20 sec stimulus, panel iv). Only a small amount of PMLC_20_ is detectable in the resting myocyte (panel vi) but this is markedly increased upon stimulation with OT (100 nM, 20 sec: panel viii).

To evaluate antibody signal specificity for GAPDH, total MLC and PMLC_20_ in the ICW, we visualized the signals using immunofluorescence microscopy (Figure 4). Slides for microscopy were prepared identically to ICW plates (see methods) to ensure that the microscopic visualizations were directly comparable to ICW measurements. The only difference in protocols was the use of secondary antibodies conjugated to Alexa-Fluor 488 for primary antibody detection for microscopy. Each slide was also stained with rhodamine-phalloidin to demonstrate filamentous actin (F-actin). In the absence of primary antibodies, there is no appreciable signal (Figure 4A panels i and ii). Using the antibody to GAPDH, there is diffuse cytosolic staining, in marked contrast to the filamentous appearance of F-actin (Figure 4A panels iii and iv). For total MLC_20_ (Figure 4A panels v and vi) the staining pattern is similar to F-actin (and in contrast to GAPDH), in keeping with the molecular co-localization of these elements of the contractile apparatus. There is no appreciable signal after pre-adsorption of the PMLC_20_ antibody with a specific blocking peptide containing phospho-Ser^19^ of MLC_20_ either in the resting state (Figure 4B panels i and ii) or after stimulation with 100 nM OT (panels iii and iv). Similarly, in our preliminary ICWs, the signal from resting and stimulated cells was reduced to background levels when the antibody toward PMLC_20_ was preadsorbed with the same blocking peptide. In the absence of the blocking peptide, there is only a faint signal in resting cells (Figure 4B panels v and vi) but this signal is rapidly inducible (20-second stimulation) in response to OT. The subcellular distribution for PMLC_20_ is identical to that of total MLC_20_ and F-actin (panels Avi, Bvii).

### Assay Precision for WB and ICW

**Figure 5:**
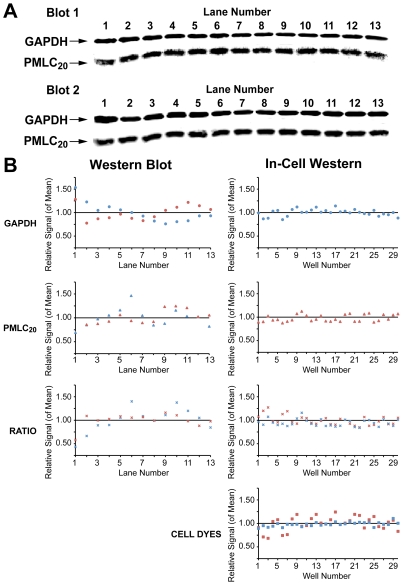
Assessment of intra-assay variability in western blots and ICW assays. **A.** WB membranes showing GAPDH and PMLC_20_ levels in 13 replicate samples in two western blots. **B.** In the panels on the left are the individual band intensities from WBs in A expressed as a proportion of the mean of the thirteen samples in each of the two blots. The data from blot 1 are shown in blue and from blot 2 in red. The data are provided for GAPDH and PMLC_20_ individually and for the ratio GAPDH/PMLC_20_. In the panels on the right are the intensities of the signals from wells distributed across ICW plates plotted as a proportion of the mean values for each of two plates (plate one in blue and plate 2 in red). The data are provided individually for GAPDH, PMLC_20_, and in the lowermost panel for the cell dyes used in data normalization. The ratios of GAPDH and PMLC_20_ to the cell dyes are also shown in red and blue, respectively.

To assess assay precision, we studied the intra-assay and inter-assay variability of each technique for GAPDH and PMLC_20_. We also evaluated the variability of normalization of PMLC_20_ to a “housekeeping” protein such as GAPDH and compared this to normalization using the cell dye markers in the ICW technique. For WB, two SDS-PAGE mini-gels were loaded with 13 replicates of 20 µg of protein/lane from the same lysate. The resultant blots (Figure 5A) were prepared simultaneously from the same electrophoresis and transfer tanks. By using secondary antibodies with distinct fluorophores, both proteins were assessed on each blot. The intra-assay variability in signal intensity across the lanes for GAPDH and PMLC_20_ is presented in Figure 5B. The intra-assay coefficients of variation (CV) for GAPDH and PMLC_20_ were 0.21 and 0.20 in blot 1, respectively, and 0.16 and for both proteins in blot 2. Blot 1 particularly emphasizes that the pattern of signal variation across the lanes was not similar for the two proteins. Hence, when the ratio of one protein to the other was calculated, the CV (0.27: Figure 5B ratio) was magnified. To determine the intra-assay CV for the ICW technique, 30 wells in each of two plates were prepared simultaneously and probed for PMLC_20_ or GAPDH. The CV for signals obtained for PMLC_20_ (Plate 1), and GAPDH (Plate 2) were 0.10 and 0.07. The CV for the cell dye estimate of cell content for plates 1 and 2 were 0.05 and 0.15, respectively. When the concentrations of PMLC_20_ or GAPDH were normalized to the cell dyes, the CV varied from 0.08 to 0.16. In summary, the intra-assay CVs for measurement of PMLC_20_ and GAPDH alone are comparable between ICW and WB but the ICW technique may have a slight advantage regarding the precision of the normalized data.

The inter-assay CVs were calculated from repeated measurement of the same samples at different times using different blots for WB or on several different plates for ICW. The inter-assay CV with the ICW technique was 0.08 for PMLC_20_ and 0.15 for the ratio of PMLC_20_ to the cell dyes. For the WB technique, the inter-assay CV was 0.16 for PMLC_20_ and 0.25 for the ratio PMLC_20_ to GAPDH.

### Measurement of Myosin Light Chain Phosphorylation in Response to a Physiological Stimulus

**Figure 6:**
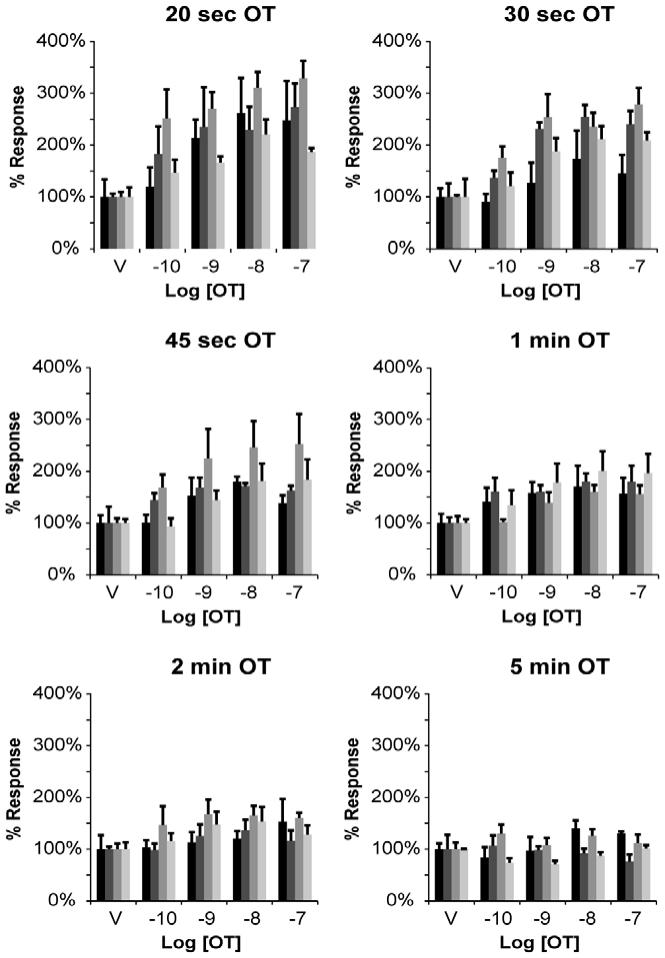
Cell density optimization for the in-cell western assay. Uterine myocytes were seeded at densities of 500 (darkest histogram in each grouping), 550, 600 and 650 (lightest histogram in each grouping) cells/mm^2^. Cells were treated with increasing concentrations of OT (10^−10^ to 10^−7^ M) for 20, 30, 45 or 60 seconds or 2 or 5 minutes (n = 4 at each time point at each concentration of OT). Concentrations of PMLC_20_ were measured using the ICW assay. PMLC_20_ levels rose rapidly (20 sec), decayed significantly by 1 min, and returned to baseline levels by 5 min. The largest amplitude concentration-dependent change in PMLC_20_ levels was measured at 20 sec and 600 cells/mm^2^.

ICWs were used to measure the effects of cell density, time course and concentration-responses for OT–induced PMLC_20_ formation. Visual inspection of myocyte cultures seeded at various densities revealed that they were nearing confluence in the range between 500–700 cells/mm^2^ (not shown). Subsequent experiments were aimed at determining the optimal cell density and time course for OT-induced PMLC_20_ formation. Stimulation with increasing concentrations and incubation times of OT for cell densities of 500, 550, 600, and 650 cells/mm^2^ demonstrated that the largest amplitude concentration-dependent rise in PMLC_20_ inducible by OT is achieved at a cell density of 600 cells/mm^2^ after treatment for 20 seconds (Figure 6). At a density of 650 cells/mm^2^ and at time points beyond 20 seconds for all densities tested, the response to OT was diminished.

**Figure 7:**
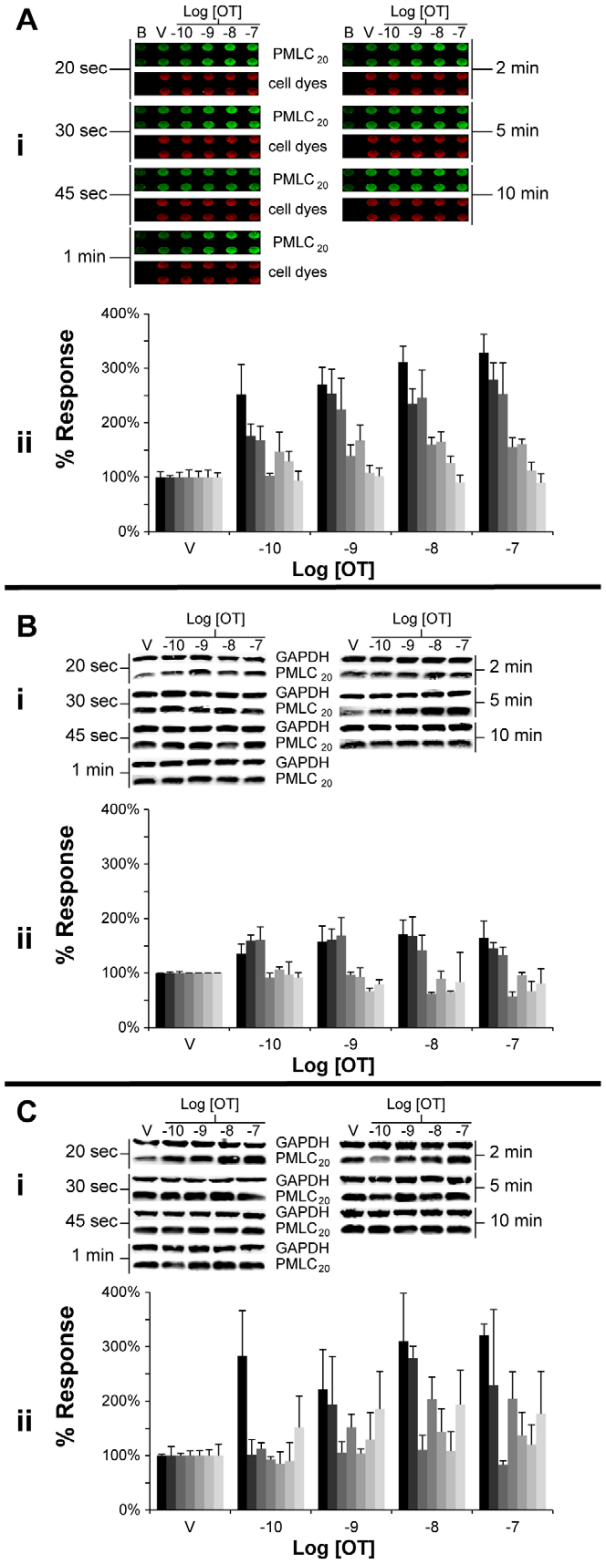
Comparison between WB and ICW for cell responses to OT. For each of the figures, the top panel (i) presents the raw data and the lower panel (ii) presents a graphical analysis of the normalized and quantified data (n = 2–4 for each histogram). For the ICW data, PMLC_20_ concentrations were normalized using the cell dyes and with the WB data, PMLC_20_ values were normalized to GAPDH. The normalized data are expressed as a percentage of the vehicle (V) controls. **A.** Data from the ICW assay. Background wells (B) had no primary antibody or cell dyes. Cells were plated at a density of 600 cells/mm^2^ and treated with vehicle or increasing concentrations of OT (10^−10^ to 10^−7^ M). The time course varied from 20 sec (darkest histogram in each grouping) through 30, 45 and 60 sec or 2, 5 or 10 minutes (lightest histogram in each grouping). **B.** The treatment and time protocols were similar to figure 7A. The protein was extracted under mild (cold PBS/Lysis Buffer) conditions and the proteins measured using western blots as indicated. **C.** Experimental protocol similar to B except that the protein extraction was performed using the more stringent (TCA/Acetone/DTT) conditions.

We next determined the time course of the concentration-response relationships of OT stimulation of uterine myocytes (at 600 cells/mm^2^) with respect to PMLC_20_ concentrations by WB and ICW under identical conditions (Figure 7). Using the ICW technique, a brisk response is measurable as early as 20 seconds after stimulation, then decays significantly by 1 min and returns to baseline levels by 5 min (Figure 7A). Qualitatively, the responses obtained by WB were similar with increases in PMLC_20_ formation acutely (<1 min) and decay of PMLC_20_ beyond 1 min. However, there were differences between the techniques with respect to quantitative data. In the first WB experiments, wet-ice cooled PBS and a lysis buffer containing phosphatase and protease inhibitors (see methods) was used to extract the protein. The maximal response to OT was measured at 171±26% of control values (Figure 7B) compared to the 329±37% using the ICW technique (Figure 7A). Since the PBS/lysis buffer is a rather mild extraction technique, we repeated the experiments using the more stringent dry-ice-cooled TCA/Acetone/DTT extraction method. This enhanced the maximal response to OT to 321±20% (Figure 7C) but the variability about the measurements appeared greater than with the ICW technique. In summary, the ICW and WB techniques yielded similar patterns of biological data with a suggestion that the ICW might have superior precision.

### Physiological Validation of Myosin Light Chain Phosphorylation Measured by In-Cell Western Assays

**Figure 8:**
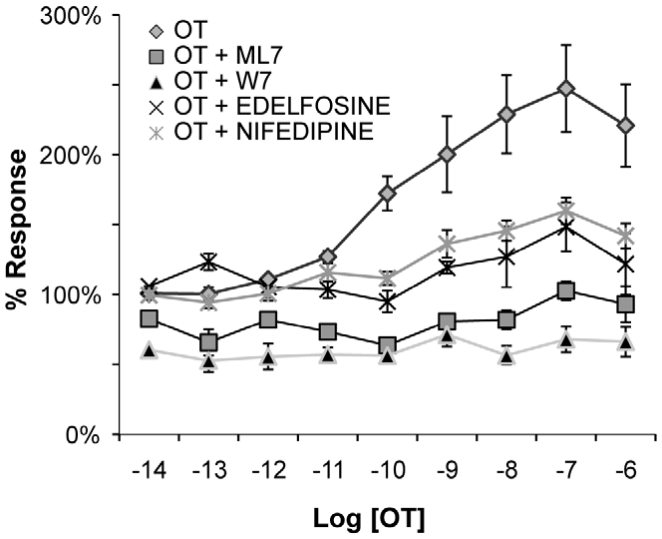
Assessment using the ICW technique of pharmacological manipulation of PMLC_20_. Uterine myocytes were seeded in microplate wells at 600 cells/mm^2^ and treated with OT (from 10^−14^ to 10^−6^ M) for 20 sec in the presence or absence of 15-minute pre-incubation with specific pharmacological agents (n = 7 separate cultures). The normalized data are expressed as a percentage of the vehicle controls. As expected, the cells demonstrated a brisk response to OT that was diminished in the presence of an inhibitor of MLCK (ML-7, 50 µM), CaM (W7, 50 µM), phosphatidyl inositol-PLC (Edelfosine, 20 µM), and L-type Ca^2+^ channels (Nifedipine, 20 µM). Relative PMLC_20_ levels (normalized to cell content) are expressed as a percentage of vehicle control. All of the pharmacological agents significantly (p<0.0001) attenuated the concentration-dependent PMLC_20_ increase induced by OT as determined by two-factor ANOVA.

To reinforce the physiological validity of the responses measured by ICW, we performed experiments in the presence or absence of pharmacologic inhibitors known to perturb the biochemical pathways mediating phosphorylation of MLC_20_. Uterine myocytes were seeded at 600 cells/mm^2^ and stimulated with OT (100 nM, 20 sec). Inhibition of MLC_20_ Kinase (MLCK) (ML7, 50 µM), calmodulin (CaM) antagonism (W7, 50 µM), inhibition of phosphatidyl-inositol-specific phospholipase C (PLC) (edelfosine, 20 µM), and inhibition of plasma membrane calcium (Ca^2+^)-channels (nifedipine, 20 µM) significantly attenuated the OT-induced rise in PMLC_20_, as assessed by two-way ANOVA (p<0.0001, Figure 8). These results indicate that the phosphorylation of MLC_20_ measured by ICW behaves as expected in accordance with current knowledge of the biochemical pathways mediating physiological PMLC_20_ responses in smooth muscle cells. [13], [14]

## Discussion

Protein phosphorylation is the most commonly studied post-translational modification due to its central role in regulating cellular physiology. PP levels are dynamically regulated in response to a plethora of biological stimuli mediated by the opposing activities of protein kinases and phosphatases. [15] These enzymes encode external and internal signals through the complement of PPs that are produced in the cell interior. Using experimental manipulations rooted in knowledge of signaling pathways, scientists can decipher the role of phosphorylated intermediates in cellular responses provided they are quantifiable. The increasing commercial availability of PP-specific antibodies has enhanced the use of WB as a standard method for this purpose.

The WB technique is a relatively labour-intensive and technically demanding assay best suited for experimental approaches that require low-throughput measurements. Our data demonstrate that the ICW technique provides signal linearity and specificity at least equal to WB. The ICW technique also provides biological response amplitudes similar to those attainable by the very robust protein collection method using dry-ice cooled TCA/Acetone/DTT, demonstrating that formalin fixation is a useful and robust method for PP signal preservation. PMLC_20_ has similarly been preserved and quantified reliably after cell fixation in other reports. [16] The ICW and WB techniques are qualitatively similar in terms of the concentration range of measurable stimulation (100 pM to 100 nM) and the time course for the rise and decay of the PMLC_20_. Both techniques were capable of measuring PMLC_20_ formation as early as 20 seconds post-stimulus. This rapid induction of PMLC_20_ agrees with other OT-response data in the literature. [17], [18] The diminished responses measured by WB using the relatively mild cold PBS/cold lysis buffer method could result from phospho-signal loss during cell lysis and harvesting, since the time necessary to complete these steps exceeds the optimal treatment length (20 sec) for a maximal response in contrast to the nearly instantaneous quenching achievable by TCA/Acetone/DTT and formalin fixation. Because we have prepared both WBs and ICWs with identical blocking and antibody incubation solutions, it is unlikely that the diminished phospho-signal measured from cold PBS/cold lysis buffer samples by WB is explained by residual phosphatase activity present in the incubation buffers. [19]

The WB technique is considered by many to be a “semi-quantitative” assay because of the relative lack of precision of the measurements. Variability may arise from the protein extraction method employed or from application of the protein to the gel. Perhaps most problematic is the transfer of the separated proteins to the blotting membrane. Uneven distribution of proteins across blotting membranes may occur since protein movement out of the polyacrylamide gel matrix occurs at unequal rates and to different extents as influenced by protein MW and solubility in the transfer buffer. [3], [4], [20], [21], [22] In particular, transfer conditions optimized for smaller proteins may not be efficient for larger proteins, and vice-versa. [3], [23] In addition, the choice of membrane, blocking and antibody solutions, and wash buffers may influence the amounts of protein retained on the membranes before antibody probing. [24], [25] These factors introduce considerable inter-assay variability, limiting the precision of the technique and ultimately complicate protein quantification via WB.

The potentially improved precision of ICW compared to WBs might be attributable to minimizing sample handling through elimination of protein extraction, electrophoresis, and protein transfer steps. Use of a calibrated digital multichannel pipette further reduces assay variability by allowing rapid treatment and quenching of many samples nearly simultaneously. In addition, the ability to take duplicate or higher number replicate measurements may further enhance precision. Though replicate measurements are feasible in the setting of WB, this is an uncommon, but perhaps advisable, practice. [26] Further, our data demonstrate that WBs may have significant lane-to-lane variability in protein transfer evidenced by inconsistencies in protein signals within and between membranes. This variability is inconsistent between two protein signals (PMLC_20_, GAPDH) and this has the potential to magnify the variability when the measurements of one protein are normalized to that of another protein. By comparison, with the ICW technique, the normalization of a protein to the cell content of the well using a combination of cell dyes appeared to give more precise normalization data (Figure 7).

In our evaluation of acute phosphorylation responses, data normalization was straightforward since protein levels were constant. However, where experiments concern longer treatment courses, it may be prudent to avoid data normalization to that of a reference protein and instead opt to use the cell dyes to avoid inaccurate interpretation of results due to changing reference protein levels. The potential for wavering levels of reference proteins has prompted others to suggest alternatives for data normalization in WBs. [26]

In the absence of purified antigen, it is impossible to obtain valid assessments of the sensitivity of assays such as WB or ICW. However, our data suggest that the ICW technique yields a precise measure of antigens down to 0.3 µg protein/well. As noted in Figure 3B, there is a high correlation with the linear plot down to 0.3 µg protein/well for all three proteins. This is in contrast to WB where the lower limit of signal discrimination for band intensities for GAPDH was 1.6 µg protein/lane. As GAPDH was the most intense signal measurable in our WB experiments, it is unlikely that PMLC_20_ or total MLC_20_ would be quantifiable below this level.

As with WB, specificity of the antibody is of paramount importance in the ICW technique. It has been suggested that the antibodies proposed for evaluation of protein antigens in fixed cells should be tested for signal intensity and specificity by WB. [27] Reliance on WB for evaluation of antibody specificity (or rather, the level of non-specificity) for what is essentially a microscopy application in ICW, presupposes that the proteome is represented identically in both techniques. However, antigen preparation is achieved using different methods for WB (reduction and linearization with DTT or 2-mercaptoethanol, and SDS) and microscopy (organic denaturation and fixation by cross-linkages). Therefore, the complement of cellular antigens is presented to the antibody in a different configuration. Hence, validation of antibody specificity for ICW using WB may prove most useful where the antibody recognizes a continuous epitope that is fully unmasked during fixation. Whether the antibody will interact with epitopes in partially denatured proteins cannot be addressed by this approach. Logically then, WB data are less appropriate for validation of antibodies raised against discontinuous epitopes encompassing amino acid residues in close proximity on the surface of a protein but separated in space in the linear amino acid sequence. This may be a more significant consideration for antibodies raised against short peptides (both mono- and polyclonal), in contrast to those (polyclonals) raised against various parts of the immunizing protein. [23], [28] One potentially less significant consideration here is that the proteome is unlikely to be represented in its entirety on a WB membrane since the gel polyacrylamide content (gel percentage) is selected so as to optimize resolution of the POI from other cellular proteins. This may result in exclusion of low MW proteins on the resultant WB if a low percentage gel is used, and may under-represent the complement of large MW proteins if a high gel percentage is selected due to their restricted mobility out of the gel during protein transfer. Thus, if one insists on WB validation of ICW antibodies, it may be useful to employ gradient gels to resolve a large MW range for this purpose. In any case, it is important to demonstrate that the antibody will detect the POI using microscopic techniques with appropriate negative controls to rule out non-specific signals. This is particularly crucial since ICWs lack the ability to separate protein signals in space as is done in WBs via SDS-PAGE.

The biggest advantages of the ICW assay compared to WB are the diminished preparation time and resource utilization as well as the potential for development of high-throughput assays with the capability to scan multiple microplates simultaneously. We also believe that the relatively small amount of cellular material in each well allows rapid quenching of treatments and preservation of the cell-interior by simple addition of fixing solution. This more uniform and exact termination of experimental treatments likely provides more precise information regarding dynamic reactions that occur in rapid time courses, exemplified by many phosphorylation reactions.

A principal prerequisite for performing ICWs is the availability of a culture method allowing for an even distribution of cells across the well surface. Cell cultures used for ICW can be adherent or non-adherent. However, if they are adherent, the cells must grow as a monolayer in culture, and, if non-adherent, must be prepared so they lie in a monolayer at the time of scanning. This will ensure a uniform signal from the well surface. In our studies, which used freshly seeded uterine myocytes, there was an optimal density of cells for a response to a physiological stimulus. The plateauing of PMLC_20_ and GAPDH signals at higher cell densities in our linearity assessment was unlikely to be due to exceeding the capabilities of the IR scanner, but rather to cell overcrowding, for two reasons. First, there were no saturated pixels in the well signals. Second, in the time course assessment we detected higher signal intensities for PMLC_20_ at 600 cells/mm^2^ in stimulated myocytes than those obtained by simply plating a higher density of cells. It is likely that the optimal density for a biological response is dependent on the cell type used, as well as other conditions, and this parameter should be assessed whenever a new cell type is studied.

Our data also demonstrate the validity of the ICW technique to monitor physiological events in a dynamic system. Phosphorylation of MLC_20_ at Ser^19^ results from influx of Ca^2+^ ions into the myocyte cytosol whether derived from the extracellular space through plasma membrane channels, or from intracellular stores whose release is triggered by the generation of inositol-trisphosphate by PLC. This Ca^2+^ influx results in activation of MLCK by the intermediary Ca^2+^ binding protein, CaM. [13], [14] We have demonstrated that pharmacological manipulation of Ca^2+^ flux, CaM activity or MLCK itself has the predicted effects on concentrations of PMLC_20_.

Clearly, there are significant limitations to the usefulness of the ICW technique. Its application is restricted to the study of proteins in cell cultures for which a specific antibody is available. It is not suitable for study of PPs or other proteins from whole tissues or in experimental situations where freshly isolated tissues are required for simultaneous measurement of physiological and biochemical endpoints. ICWs are also restricted to the study of proteins present in high enough levels to be detectable from the cellular material in a single well, as it is not possible to concentrate low-abundance proteins. In contrast, WBs can be preceded by a protein-concentrating or protein-purifying technique such as immunoprecipitation. Another disadvantage of ICWs is that they do not allow determination of the stoichiometry of phosphorylation, such as can be obtained using urea/glycerol PAGE [29] or via phos-tag [10] SDS-PAGE. Further, unless one has access to an infrared microscope, ICWs do not permit routine identification of the proteins yielding the measured signal, which is verified in each WB membrane using the MW marker. Lastly, whereas WBs are routinely used to analyze samples that have been frozen in storage, ICWs are unlikely to be useful in the same way since ice crystals formed during freezing may damage the integrity of the cellular structures necessary to obtain reliable data by this method. Therefore, in our experience, ICWs should be used to analyze microplates that have been stored only briefly (a few days) at 4°C. We have not formally addressed the length of time that protein phosphorylation is preserved in ICWs, and for this reason we proceeded with the preparation of microplates immediately after cell fixation. Our general observations are that some phospho-antigens are stable over several days (perhaps weeks) after fixation and storage at 4°C in PBS, but others decay more rapidly. Thus the window of time for plate development must be determined empirically for each PP.

In conclusion, we have provided validation of the use of the ICW technique to assess rapid phosphorylation events in uterine myocytes. It is likely that this technique is equally applicable to many other cell types to assess many other physiological reactions. We have demonstrated that there may be advantages of the ICW to conventional WB techniques with respect to sensitivity and precision and, depending on the proper validation of the antibody, the two techniques are of similar specificity. The major advantage of the ICW assay over WB is the relative ease of preparation of the samples and the potential for high-throughput data production. The increased relative cost of ICWs due to the need for lower dilutions of some antibodies is offset by the much higher throughput capacity in addition to other savings in reagents by elimination of several preparative steps from the WB protocol. Our data demonstrate that the technical capabilities of ICWs meet or exceed the standard set by NIR fluorescent WBs. Considering the technical aspects of the two procedures, we conclude that the benefits of ICWs are generalizable irrespective of the protein target of interest.

## Materials and Methods

### Ethics Statement

The protocol to obtain biopsies from the lower segment of the uterus at the time of cesarean section was reviewed and approved by the Human Ethics Review Board of the University of Alberta and the Ethics Review Board of Capital Health, the provider of health services in the region. A research nurse at the Royal Alexandra Hospital in Edmonton obtained informed and written consent from each patient.

### Primary Myometrial Cell Cultures

Myometrial biopsies were obtained from the lower segment of the uterus from non-labouring patients at term (37–40 weeks) gestation during elective caesarean section. Biopsies were cut into small pieces with ethanol-sterilized tools in a sterile cell culture hood, then incubated in filter sterilized (0.22 µm) Hanks Balanced salt solution (HBSS, Gibco, (Invitrogen, Carlsbad, CA)) containing 1% antimycotic/antibiotic (Gibco; 10,000 U/mL of penicillin, 10,000 µg/mL of streptomycin, 25 µg/mL of amphotericin B), 2 mg/mL collagenase (Sigma-Aldrich, St. Louis, MO), and 200 ng/mL DNase I (Roche, Laval, QC) in a 50 mL conical tube (Corning) in a total volume of 10 mL of the prepared HBSS. These tubes were incubated for 1 hr at 37°C with shaking. After 1 h, the debris was allowed to settle and the supernatant (containing fibroblasts) was discarded. The remaining tissue was incubated in another 10 mL of prepared HBSS at 37°C while shaking, for 4 hrs to overnight (O/N). The dispersed cell mixture was filtered through a 100 µm cell strainer, centrifuged at 2000×*g* for 5 min, and washed twice with sterile phosphate buffered saline (PBS). Isolated uterine myocytes were grown in Dulbecco's modified Eagle's medium (DMEM, Gibco) supplemented with 10% fetal bovine serum (FBS, Gibco) and 1% antimycotic/antibiotic (Gibco) at 37°C in humidified 5% CO_2_/95% air. Cell cultures were grown to 80–100% confluence in a single well of a 6-well plate post-isolation from tissue biopsies, then in T25 and T75 flasks (Ultident, St. Laurent, QC). Incubation at 37°C with trypsin (0.05%, Gibco) was used to free cells from the substrate. An excess of culturing medium was used to quench the trypsinization reaction. Cells were monodispersed by vigorous pipetting, counted using a hemocytometer and then plated at the desired cell density onto sterile black-walled 96 well (Greiner Bio-One, Monroe, NC) microplates for ICW, in sterile 6-well plates (Corning, Lowell, MA) for WB, or on 8-chamber slides (VWR Int., USA) for fluorescence microscopy experiments. Prior to experiments, the cells were washed once with pre-warmed DMEM containing no additives, and then placed into the incubator in DMEM without additives for 2–4 hrs. All stimulants, pharmacologic agents, and drug diluents (DMSO) were diluted in DMEM without additives to the final concentration prior to experiments. Oxytocin (OT), ML-7 (MLC_20_ Kinase inhibitor), W7 (calmodulin antagonist), edelfosine (phospholipase C inhibitor), and nifedipine (calcium channel blocker) were all acquired from Calbiochem.

### Total Protein Extraction

For WB, total cellular protein was extracted by two methods. Method 1: cells were washed after treatment with ice-cold PBS followed by addition of ice-cold lysis buffer containing 50 mM Tris-HCl pH 8.0, 137 mM NaCl, 5 mM EDTA, 0.1% Triton X-100, 1 mM PMSF, 1X protease inhibitor cocktail (Sigma-Aldrich, Final concentrations: 1.04 mM ASBSF, 0.8 µM Aprotinin, 21 µM Leupeptin, 36 µM Bestatin, 15 µM Pepstatin A, 14 µM E-64), and 1X phosphatase inhibitor cocktail (PhoSTOP, Roche) for detection of PPs. Cells were freed from the substrate using a cell scraper and collected into wet-ice-cooled microfuge tubes, then snap frozen in liquid nitrogen and stored at −80°C for later use. Protein concentrations were determined by combining 1 part sample to 99 parts Precision Red™ advanced protein measurement reagent (ADV02, Cytoskeleton, Denver, CO, USA) according to the manufacturer's instructions. Method 2: after treatment, cells were lysed in an acetone solution containing 10% trichloroacetate (TCA) 10 mM dithiothreitol (DTT), and protease inhibitors as indicated above. This solution was pre-cooled in a dry-ice methanol bath before addition to the culture plate. Cells were immediately scraped from the substrate as above, and then collected into wet-ice-cooled microfuge tubes. These tubes were centrifuged at 18,000×*g* for 30 min at 4°C, the supernatant was removed, and the pellet was resuspended by vortexing in acetone containing 10 mM DTT, and allowed to sit O/N. The next day, tubes were centrifuged at 18,000×*g* for 15 min at 4°C. The washing solution was removed and the pellets were allowed to air-dry for 20–30 min. The dried pellets were resuspended in 60 µL of 1X SDS-PAGE loading buffer (see below) and incubated at 65°C for 10 min before storage or loading 17.5 µL from each onto SDS-PAGE minigels.

### SDS-PAGE and Protein Transfer

Total protein was prepared for electrophoresis by adding 5X loading buffer (Final concentrations: 10% glycerol, 3% SDS w/v, 85 mM Tris HCl pH 6.8, 85 mM dithiothreitol, 0.1% bromophenol blue) and boiling for 10 minutes. Approximately 25 µg/lane of protein was loaded onto 15% or 18% Tris-Glycine-SDS minigels, or alternatively, onto 12% gels with or without 30 µM phos-tag [10] reagent (NARD Institute, Ltd., www.phos-tag.com) combined with 60 µM MnCl_2_ to demonstrate shifts in mobility for PPs. SDS-PAGE was carried out in 1X SDS running buffer (10X Tris-Glycine-SDS buffer, BioRad, Hercules, CA, USA) using a constant current of 15 mA for the stacking (4.2% acrylamide, pH 6.8) gel and then a constant 25–30 mA for the separating (15% or 18% acrylamide, pH 8.8) gel in a Mini-PROTEAN 3 electrophoresis apparatus (BioRad), according to the Laemmli method. [30] Precision Plus Protein all blue standard (2.5 µL/lane, BioRad) was loaded on each gel for visualization in the 700-channel of the Odyssey® imager. The gel was transferred to nitrocellulose (Li-COR, Biosciences, Lincoln, NE, USA) at 4°C in a Mini-Trans-Blot cell (BioRad) apparatus using a buffer containing 20% MeOH and 1X Tris-Glycine (10X Tris-Glycine, Li-COR) for 1.5 hrs at 100 V. Gels containing phos-tag and MnCl_2_ were incubated at room temperature (R/T) for 15–20 min in transfer buffer of the same composition supplemented with 2 mM EDTA to chelate and remove Mn^2+^ ions, then allowed to equilibrate in transfer buffer without EDTA for an additional 15–20 min prior to transfer.

### Western Immunoblotting

After transfer, membranes were rinsed briefly in PBS. Nitrocellulose membranes were placed into Odyssey® blocking buffer (OBB, Li-COR) diluted 1∶1 with PBS immediately and blocked for 1 hr at R/T. Primary antibodies were diluted in OBB combined 1∶1 with PBS, containing 0.1% Tween-20, and incubated O/N at 4°C. The final antibody concentrations used were 1∶1000 for GAPDH (mouse MAb, Santa Cruz), 1∶500 for MLC_20_ (Rabbit PAb, Cell Signaling), 1∶200 for PMLC_20_ (rabbit PAb, Cell Signaling), and 1∶200 for PPMLC_20_ (diphospho Thr^18^ Ser^19^, rabbit PAb, Santa Cruz). After primary antibody incubations, membranes were washed 3X (10 min each) with PBS containing 0.1% Tween-20 (PBS-T) at R/T on a bench top shaker. Secondary antibodies conjugated to IRDye 800CW (Li-COR) or Alexa Fluor 680 (Molecular Probes, Invitrogen) were diluted to 1∶20,000 in OBB combined 1∶1 with PBS, containing 0.1% Tween-20 and 0.01% SDS. Membranes were incubated with secondary antibody solutions for 1.5 hrs at R/T and in the dark on a bench top shaker. After secondary antibody incubations, the membranes were washed 3X (10 min each) with PBS-T at R/T on a bench top shaker in the dark, then briefly rinsed in PBS before scanning. Membranes were scanned and analyzed using an Odyssey® IR scanner using Odyssey® imaging software 3.0. Scan settings were medium or high image quality, 169 µm resolution, intensity 3.0–5.0 for both channels with no offset. Antibody signals were analyzed as integrated intensities of regions defined around the bands of interest in either channel.

### In-Cell Westerns

Cells were plated in sterile black-walled 96 (half-area) (Greiner Bio-One, Monroe, NC) plates at the desired density then placed at R/T for 30–60 minutes before transfer to the incubator to reduce excessive settling at the well edges. [31] The plates were incubated O/N at 37°C as indicated above. Prior to treatment, all wells were washed with DMEM, and incubated in DMEM for 2–4 hours before beginning the experiments. Cell treatments were dispensed with a calibrated digital multichannel pipette with a repeater function to ensure accuracy in treatment timing. After treatment, cells were fixed immediately by adding concentrated formalin to a final concentration of 3.7% formaldehyde and incubating at R/T for 10 minutes. After fixation, the plates were washed with PBS then permeabilized by washing 3X (10 min each) on a bench top shaker with PBS containing 0.1% Triton-X-100, and then rinsed once with PBS. Cells were blocked using 20 µL/well OBB for 1 hr at R/T. Primary antibodies were prepared as for WB and the plates were incubated O/N at 4°C using 20 µL/well. The final antibody concentrations used were 1∶500 for GAPDH (mouse MAb, Santa Cruz) and 1∶200 for PMLC_20_ (Rabbit PAb, Cell Signaling). After primary antibody incubations, plates were washed 3X (10 min each) with 100 µL/well PBS-T at R/T on a bench top shaker. Secondary antibodies were prepared as for WB with a few modifications: IRDye 800CW conjugates of Goat-anti-rabbit-IgG (Li-COR), goat-anti-mouse-IgG (Li-COR) were used at 1∶1000 dilution for detection of antibody targets in the 800-channel (green pseudocolor). 800-channel antibody signals were normalized to the 700-channel (red pseudocolor) signals derived from Alexa-Fluor 680 conjugated goat-anti-mouse-IgG secondary at 1∶1000 (Molecular Probes, Invitrogen), or DRAQ5 (Biostatus Ltd., Leicestershire, UK) combined with Sapphire700 (Li-COR) at 1∶10,000 and 1∶1000 respectively. Plates were incubated with 20 µL/well secondary antibody solutions for 90 min at R/T in the dark. Background control wells were prepared by omitting primary antibodies, DRAQ5 and Sapphire700 (i.e. secondary only). After secondary antibody incubations, plates were washed 3X (10 min each) with PBS-T at R/T on a bench top shaker in the dark, and then filled with 50 µL/well PBS to reduce surface disturbances when scanning. Plates were scanned and analyzed using an Odyssey® IR scanner using Odyssey® imaging software 3.0. Scan settings were medium or high image quality, 169 µm resolution, intensity 5.0 for the 700-channel, and 7.0 for the 800-channel with an offset of 4.0 mm. For signal quantification, antibody signals were analyzed as the average 800-channel integrated intensities from duplicate wells normalized to the 700-channel signal integrated intensity to correct for well-to-well variations in cell number. Results are expressed as percent relative responses (means ± standard errors of the mean) compared to vehicle-treated controls.

### Microscopy

8-chamber sterile culture slides (BD Falcon) were seeded, cultured, treated, fixed, and prepared for fluorescence or brightfield microscopy exactly as culture plates for ICWs, with the exception of utilizing a lower dilution of primary antibodies (1∶50 MLC_20_ (Rabbit PAb, Cell Signaling), 1∶50 PMLC_20_ (Rabbit PAb, Cell Signaling), 1∶200 GAPDH) and DyLight 488-conjugated goat-anti-rabbit-IgG (Cell Signaling) and goat-anti-mouse-IgG (Cell Signaling) antibodies at a dilution of 1∶100. After secondary antibody application, cells were rinsed 3X in PBS-T and then incubated with 50 nM rhodamine-phalloidin (Cytoskeleton, Denver, CO, USA) for 10 min at R/T to reveal F-actin fiber distribution. Slides were rinsed 3X in PBS then mounted in VECTASHIELD (Vector Laboratories, Burlington, ON, Canada) mounting medium containing DAPI to detect nuclei. Slides were allowed to rest for 60 min in the dark and stored at 4°C if necessary before visualizing under an Olympus IX81 fluorescent microscope (Carson Scientific Imaging Group; Ontario, Canada) using Slidebook 2D, 3D Timelapse Imaging Software (Intelligent Imaging Innovations Inc.; CO, U.S.A.).

### Statistical Analysis

The results were expressed using means (±S.E.M.). Statistical analysis was performed using One-way ANOVA to compare the means of 3 or more groups at a time in OT-induced MLC_20_ phosphorylation time course studies. Two-factor ANOVA was used to determine if pharmacologic inhibitors had an effect on the OT-induced MLC_20_ phosphorylation. A P-value of 0.05 was used to establish statistical significance.
